# Early Fault Diagnosis for Planetary Gearbox Based Wavelet Packet Energy and Modulation Signal Bispectrum Analysis

**DOI:** 10.3390/s18092908

**Published:** 2018-09-01

**Authors:** Junchao Guo, Zhanqun Shi, Haiyang Li, Dong Zhen, Fengshou Gu, Andrew D. Ball

**Affiliations:** 1 School of Mechanical Engineering, Hebei University of Technology, Tianjin 300401, China; jc_guo12@163.com (J.G.); z_shi@hebut.edu.cn (Z.S.); 2 Centre for Efficiency and Performance Engineering, University of Huddersfield, Huddersfield HD1 3DH, UK; haiyang.li@hud.ac.uk (H.L.); f.gu@hud.ac.uk (F.G.); a.ball@hud.ac.uk (A.D.B.)

**Keywords:** Wavelet Packet Energy (WPE), Modulation Signal Bispectrum (MSB), time-frequency subspaces, planetary gearbox

## Abstract

The planetary gearbox is at the heart of most rotating machinery. The premature failure and subsequent downtime of a planetary gearbox not only seriously affects the reliability and safety of the entire rotating machinery but also results in severe accidents and economic losses in industrial applications. It is an important and challenging task to accurately detect failures in a planetary gearbox at an early stage to ensure the safety and reliability of the mechanical transmission system. In this paper, a novel method based on wavelet packet energy (WPE) and modulation signal bispectrum (MSB) analysis is proposed for planetary gearbox early fault diagnostics. First, the vibration signal is decomposed into different time-frequency subspaces using wavelet packet decomposition (WPD). The WPE is calculated in each time-frequency subspace. Secondly, the relatively high energy vectors are selected from a WPE matrix to obtain a reconstructed signal. The reconstructed signal is then subjected to MSB analysis to obtain the fault characteristic frequency for fault diagnosis of the planetary gearbox. The validity of the proposed method is carried out through analyzing the vibration signals of the test planetary gearbox in two fault cases. One fault is a chipped sun gear tooth and the other is an inner-race fault in the planet gear bearing. The results show that the proposed method is feasible and effective for early fault diagnosis in planetary gearboxes.

## 1. Introduction

Planetary gearboxes are widely used in wind turbines and other large machines and are an important feature of mechanical transmission systems. As planetary gearboxes usually work under heavy loading and severe environment, their failure may cause accidents and result in higher maintenance costs [1,2]. Early diagnosis of planetary gearbox contributes to the normal operation of mechanical equipment [3,4]. One of the most widely used methods to diagnose planetary gearbox failure is vibration-based analysis, because it is easily measured and contains plenty of fault information [5]. By applying advanced signal processing methods, one can extract the features of the faulty components from the measured vibration signals [6,7]. Therefore, it is important to investigate an effective and feasible method to accurately extract fault features from vibration signals to detect planetary gearbox failures.

At present, fault diagnosis methods could mainly be divided into two categories as model-based and data-driven [8]. The model-based method requires extensive system knowledge and related dynamics, which is difficult to implement in practically complex applications [9]. By contrast, the data-driven method has been widely used in rotating machinery fault diagnosis because detailed physical system dynamic knowledge is not required [10,11,12]. Moreover, many regular analysis methods for fault feature extraction have been applied to planetary gearbox fault diagnosis [13,14,15], such as envelope analysis (EA), maximum correlated kurtosis deconvolution (MCKD), Wigner–Viller distribution (WVD), empirical mode decomposition (EMD), etc. Despite the above method having been widely applied to the fault diagnosis of planetary gearboxes, all methods have limitations. For example, EA needs to choose center frequency and bandwidth for the bandpass filter in advance [13]; MCKD is restricted by its parameter selection, which will affect the denoising result [14]; WVD is subjected to cross term interference [15]; and EMD is often affected by boundary effects and modes mixing [16,17].

Different from these regular analysis methods Wavelet Packet Energy (WPE) is an alternative method formed by linear combinations of usual wavelet functions [18]. WPE can be used to reveal the energy distribution information of the vibration signal in each time-frequency subspace and highlight the energy concentrated subspaces. According to the failure mechanism of bearing, the energy level of the vibration signals related to the fault will be increased and concentrated in a certain frequency band when a fault occurs [19]. Therefore, the frequency band with high energy level in the divided time-frequency subspaces which are decomposed by WPE is more related to the fault and can be used to indicate the fault features. WPE has received much attention in the area of machinery fault diagnosis [20,21,22,23]. He proposed a novel method to probe the WPE flow characteristics of vibration signals by using the manifold learning technique [20]. Wang et al. proposed a novel method based on WPE and manifold learning to extract the weak signature and outperform the Kurtogram-based methods [21]. Gómez et al. calculated WPE as an effective fault feature to detect railway axle cracks, then classify and identify fault features through artificial neural network (ANN) [22]. Zhou et al. proposed a hybrid method that the WPE value of the first several intrinsic mode functions (IMFs)IMFs decomposed by EMD is taken as the fault feature, and then classified and identified by support vector machine (SVM)SVM [23]. The results of the above studies have indicated that WPE could effectively diagnose machine failures. However, the reconstructed signal from the relatively high energy vector of the WPE matrix still has noise and modulation components.

Recently, Modulation Signal Bispectrum (MSB) has appeared in fault diagnosis thanks to its ability to effectively reveal modulation characteristics and suppress noise. Gu et al. explored a novel analysis method for motor broken rotor bar diagnosis using the MSB-based motor current signal analysis (MCSA) [24]. Zhang attested MSB-MCSA is an effective and precise method to monitor gear wear [25]. Tian demonstrated that an MSB-based method can produce more exact and robust test results for the fault diagnosis of bearings in some scenarios [26]. However, the frequency implementation of MSB could limit MSB noise suppression performance because of spectral smearing.

In this paper, a new analysis method based on WPE and MSB for planetary gearbox fault diagnosis is proposed. First, the signal energy will be decomposed into different time-frequency subspaces by WPT, forming a WPE flowing from the low layer to the high layer. Then, the energy concentrated time-frequency subspaces are selected from WPE, and an inverse WPT is applied to the selected bands to obtain a reconstructed signal. Finally, MSB is used to suppress the noise and demodulate the modulation components from the reconstructed signal, and then extract the fault characteristic frequency for the fault diagnosis of planetary gearboxes. The results indicate that the proposed approach performs excellently in the early extraction of planetary gearbox fault characteristics.

The rest of the paper will be organized as follows. Section 2 briefly introduces the theoretical background on the WPE feature extraction. Section 3 describes the fundamental theory of MSB and its application on sideband estimation. Section 4 presents the process of the proposed method based on WPE and MSB for planetary gearbox fault diagnosis. The proposed method is verified by two planetary gearbox application cases in Section 5. The conclusions are given in Section 6.

## 2. Wavelet Packet Transform and Energy Feature Extraction

Let $V_{0,0}$ is a vector space of $R^{n}$, which is the node 0 of in the parent tree. The vector space could be split into two mutually orthogonal subspaces at each level and be expressed as [27]:

(1) $$V_{j,k}=V_{j+1,2k}⊕U_{j+1,2k+1}$$

where j and k are the layer of the tree and index of the node in layer j respectively, and $k=0,\ldots ,2^{j}-1$.

The decomposition process of WPT is depicted as follows. First, the WP function $W_{j,k}^{n}\left(t\right)$ can be expressed as [28]:

(2) $$W_{j,k}^{n}\left(t\right)=2^{j/2}W^{n}\left(2^{j}t-k\right)$$

where j indicates the scale, k represents the translation parameters, and n=0,1,…. When j=k=0, the first two wavelet packet functions Φ(t) and Ψ(t) are the scaling function and mother wavelet function respectively, and they can be expressed as follows:

(3) $$W_{0,0}^{0}\left(t\right)=\Phi (t)$$

(4) $$W_{0,0}^{1}\left(t\right)=\Psi (t)$$

The remaining WP functions of n=2,3,… can be defined by the following recursive relationships:

(5) $$W^{2n}\left(t\right)=\sqrt{2}\underset{k}{\sum }h\left(k\right)W_{1,k}^{n}\left(2t-k\right)$$

(6) $$W^{2n+1}\left(t\right)=\sqrt{2}\underset{k}{\sum }g\left(k\right)W_{1,k}^{n}\left(2t-k\right)$$

where $h\left(k\right)=1/\sqrt{2}\langle \varphi \left(t\right),\varphi \left(2t-k\right)\rangle$ is the filter coefficient of low-pass filter and $g\left(k\right)=1/\sqrt{2}\langle \Psi (t),\;\Psi \left(2t-k\right)\rangle$ is the filter coefficient of high-pass filter. They are orthogonal to the relationship $g\left(k\right)={\left(-1\right)}^{k}h\left(1-k\right)$. Here, 〈.,.〉 indicates the inner product operator. The wavelet packet coefficients $Q_{j,k}^{n}$ can be calculated through the signal x(t) and the wavelet packet functions $W_{j,k}^{n}$, as [29]:

(7) $$Q_{j,k\;}^{n}=\langle x,W_{j,k}^{n}\left(t\right)\rangle =\int_{-\infty }^{+\infty }x\left(t\right)W_{j,k}^{n}\left(t\right)dt$$

where $Q_{j.k}^{n}$ and *k* are the *n*th set of WP coefficients at the *j*th scale parameter and translation parameter, respectively.

As a result, at the *^j^*th layer, the signal x(t) is separated into $2^{j}$ packets with the order $n=1,2,\ldots ,2^{j}$. The nodes can be indexed by (j,n) in the binary tree structure, and the tree structure of the WPT comprises nodes with equal bandwidth for each scale parameter *j*. After reconstructing the coefficients at each node layer by layer in the tree structure, a waveform feature space is obtained.

For a discrete signal x(t) with $N\left(N=2^{n_{0}}\right)$ points, the reconstructed signal of wavelet coefficients in node can be expressed using $\left\{R_{j}^{n}\left(k\right),k=1,2,\ldots ,2^{n_{0}}\right\}$. If assuming the WP node (j,n) is a container in the area of $2^{n_{0}}$, the waveform featured distributed in the container can be represented by $R_{j}^{n}$. Then the waveform feature (WF) space for the (j,n)th layer can be expressed as follows [21]:

(8) $$WF_{j}=\{\begin{array}{c} x(t)\;,j=0\\ \left[\overset{n=1,2,\cdots ,2^{j}}{\overset{︷}{{\left[R_{j}^{1}\right]}_{2^{n_{0}}\times 1}\ldots {\left[R_{j}^{n}\right]}_{2^{n_{0}}\times 1}\ldots {\left[R_{j}^{2^{j}}\right]}_{2^{n_{0}}\times 1}}}\right] \end{array}\in R^{2^{n_{0}}\times 2^{j}},j\in \left(1,J\right)$$

Put $WF_{j}$ from layer 0 to J, then the *WF* is expressed as follows:

(9) $$\mathrm{WF}={\left[\overset{n=1,2,\cdots ,2^{j}}{\overset{︷}{{\left[WF_{0}\right]}_{2^{n_{0}}\times 2^{0}}\ldots {\left[WF_{n}\right]}_{2^{n_{0}}\times 2^{n}}\ldots {\left[WF_{0}\right]}_{2^{n_{0}}\times 2^{J}}}}\right]}^{T}\in R^{D\times 2^{n_{0}}}$$

where $D=2^{0}+2^{1}+\ldots +2^{J},$ which is the dimension of the WF space.

## 3. Modulation Signal Bispectrum

For a discrete time series x(t) with corresponding discrete fourier transform X(f), the modulation signal bispectrum (MSB) can be expressed in the frequency domain as [24]:

(10) $$B_{MS}\left(f_{1},f_{2}\right)=E\langle X\left(f_{1}+f_{2}\right)X\left(f_{1}-f_{2}\right)X^{*}\left(f_{1}\right)X^{*}\left(f_{1}\right)\rangle$$

where $B_{MS}\left(f_{1},f_{2}\right)$ is the bispectrum of signal x(t), E<> indicates the expectation operator, $f_{1}$ is carrier frequency, $f_{2}$ is the modulating frequency, $\left(f_{1}+f_{2}\right)$ and $\left(f_{1}-f_{2}\right)$ are the higher and lower sideband frequencies. The bispectral peak of $B_{MS}\left(f_{1},f_{2}\right)$ can represent the modulation characteristics.

To quantify the sideband amplitude more accurately, the MSB is modified via removing the effect of $f_{1}$ through magnitude normalization. The MSB sideband estimator is defined as [26]:

(11) $$B_{MS}^{SE}\left(f_{1},f_{2}\right)=\frac{B_{MS}\left(f_{1},f_{2}\right)}{\sqrt{\left|B_{MS}\left(f_{1},0\right)\right|}}$$

where $B_{MS}\left(f_{1},0\right)$ represents the squared power spectrum estimation at $f_{x}=0$.

In order to obtain more robust results, the estimator is improved based on the average of several suboptimal MSB slices, and can be denoted by:

(12) $$B\left(f_{2}\right)=\frac{1}{N}\overset{N}{\underset{n=1}{\sum }}B_{MS}^{SE}\left(f_{1}^{n},f_{2}\right)\;(f_{2}>0)$$

where *N* indicates the whole number of selected $f_{1}$ suboptimal slices, the number of which relies on the significance of the peaks themselves.

To get suboptimal $f_{1}$ slices, the $B_{MS}^{SE}\left(f_{1}^{n},f_{2}\right)$ is defined as the compound MSB slice $B\left(f_{1}\right)$, calculated by averaging the main MSB peaks in the incremental direction of the $f_{2}$:

(13) $$B\left(f_{1}\;\right)=\frac{1}{M-1}\overset{M}{\underset{m=2}{\sum }}B_{MS}^{SE}\left(f_{1,}m\Delta f\right)$$

where Δf denotes the frequency resolution in the $f_{2}$ direction.

## 4. The Proposed Method Based on WPT and MSB

**Figure 1:**
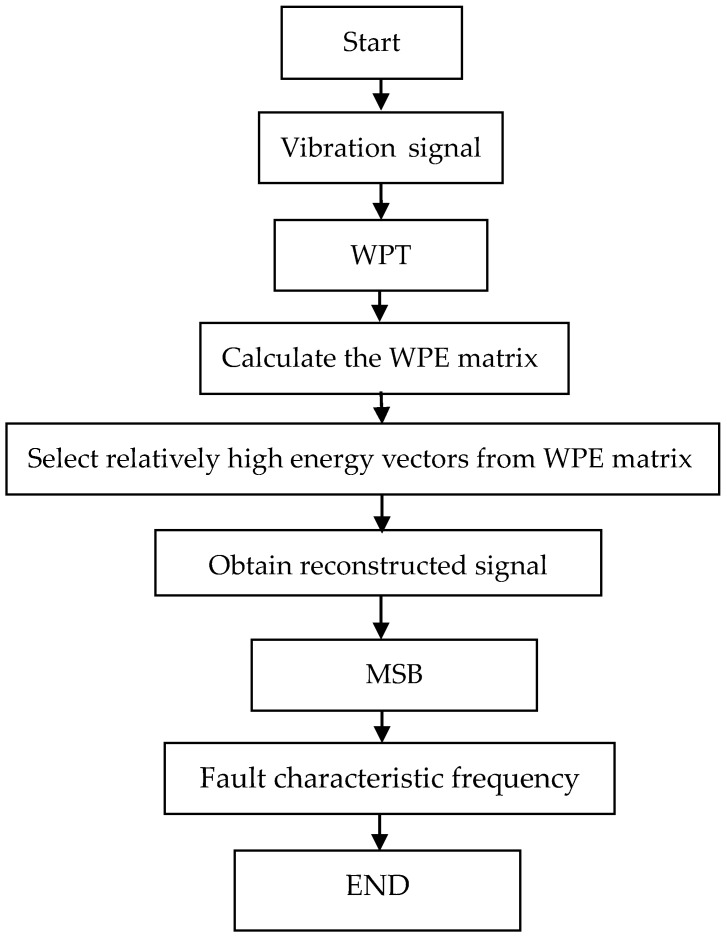
Flowchart of the proposed method.

Based on the advantages of WPE and MSB, the WPE–MSB for fault diagnosis of planetary gearbox is proposed. The scheme of the proposed method is briefly illustrated in Figure 1 and depicted as follows:

Step 1: Carry out WPT to process the vibration signal in order to separate it into different time-frequency subspaces.
Step 2: Calculate the WPE matrix in time-frequency subspaces.
Step 3: Select relatively high energy vectors from the WPE matrix.
Step 4: Apply an inverse WPT to the selected bands to obtain reconstructed signal.
Step 5: Calculate MSB of the reconstructed signal to extract the fault characteristic frequency.

## 5. Experimental Verification

### 5.1. Experimental Setup 

**Figure 2:**
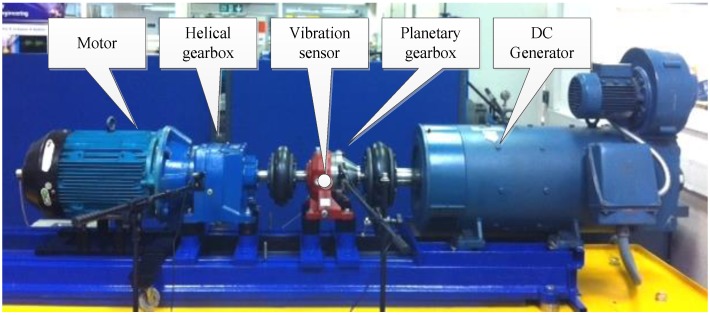
The planetary gearbox test rig.

**Figure 3:**
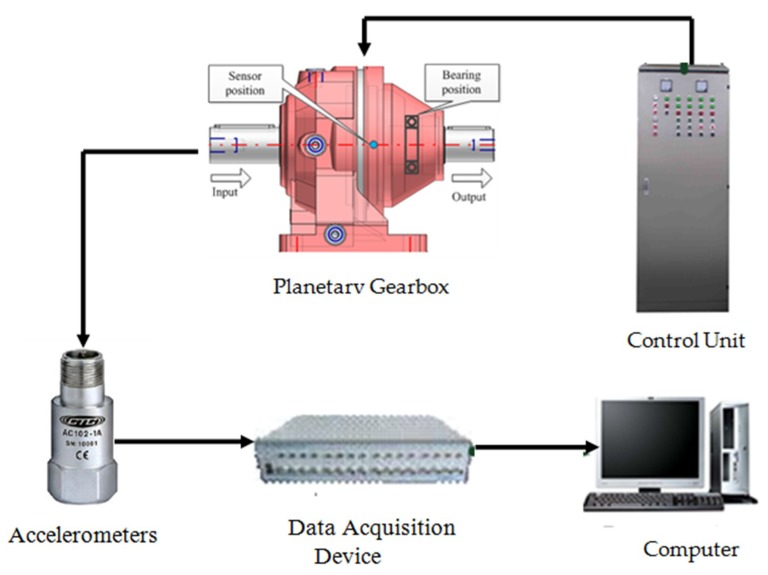
The measurement system.

**Figure 4:**
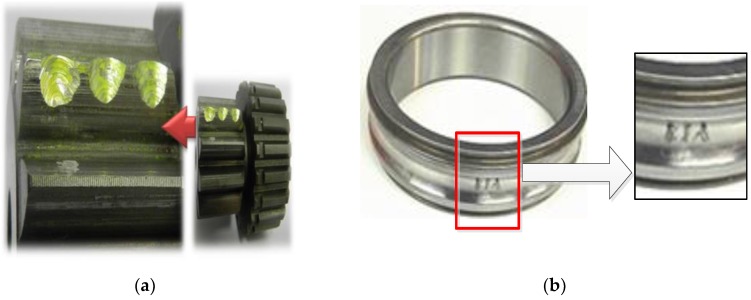
The fault modes: (**a**) sun gear chipped; (**b**) planetary gear bearing with inner-race fault.

The vibration signal was recorded based on the planetary gearbox test rig shown in Figure 2. The rated torque of the test planetary gearbox was 670 Nm, and the maximum input speed was 2800 rpm with a resulting output speed of 388 rpm. The accelerometer with a sensitivity of 28.7 $\mathrm{mV}/{\mathrm{ms}}^{-2}$ was mounted on the outer housing of the ring gear along the position of the test planetary gear bearing. The collected data were sampled at 96 kHz with a resolution of 24 bits in the measurement system as shown in Figure 3. The fault modes include sun gear chipped and planetary gear bearing with inner-race fault, as illustrated in Figure 4a,b, respectively. The structural and kinematical parameters of the test planetary gearbox and planetary gear bearing are listed in Table 1 and Table 2.

### 5.2. The Fault Diagnosis of the Sun Gear Chipped Tooth

**Figure 5:**
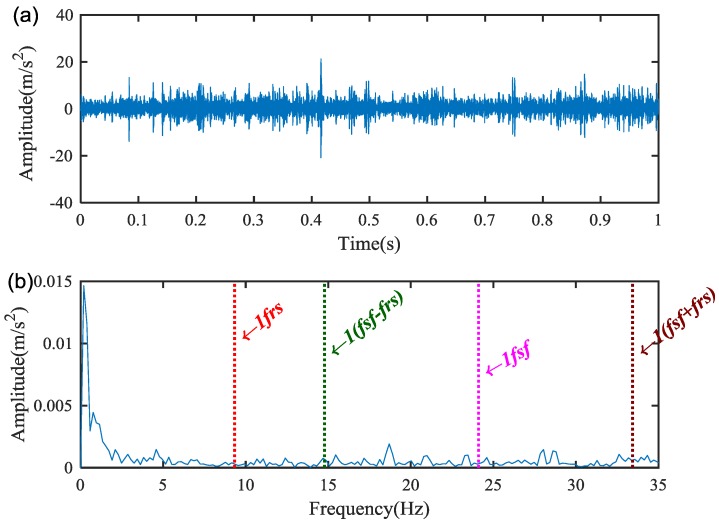
The raw vibration signal of the sun gear chipped: (**a**) the waveform, and (**b**) frequency spectrum.

The measured vibration signal under the sun gear chipped tooth fault and the corresponding spectrum are shown in Figure 5a,b. It is difficult to find the characteristic frequency of the sun gear chipped due to the heavy noise.

**Figure 6:**
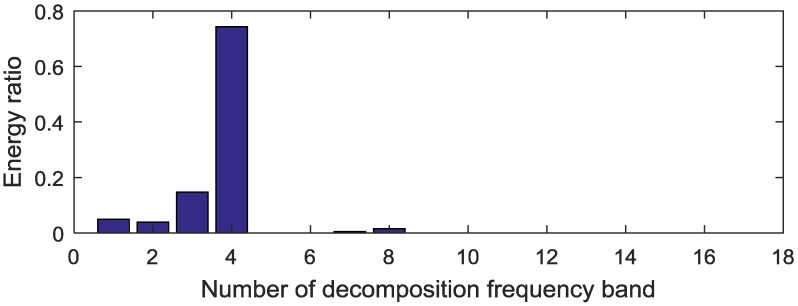
Energy distribution proportional histogram of the sun gear chipped.

**Figure 7:**
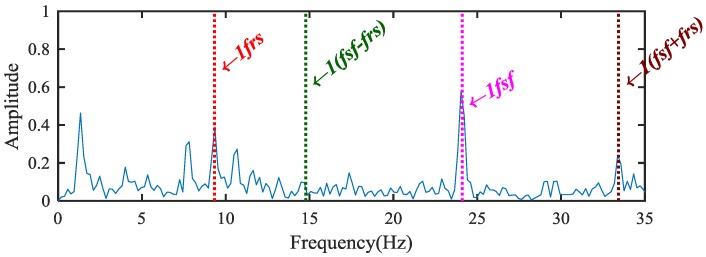
The envelope spectrum of the reconstruction signal.

The proposed method is used to analyze the measured vibration signal in Figure 4a. First, the WPT method is applied to extract fault features from the separated signals. The db 10 wavelet function is used for 4-layers decomposition, then 16 decomposition bands are obtained [30,31] and the frequency range of the 4-layers wavelet packet are shown in Table 3. Then the WPE matrix is calculated, and the energy distribution proportional histogram of the vibration signal of the sun gear chipped is shown in Figure 6. It is clear that the ratios of the energy distribution are higher at the node [4,2] and [4,3] than other nodes. Therefore, the above two bands are selected to reconstruct a signal. Envelope analysis is then applied to process the reconstructed signal, and the obtained normalized envelope spectrum is show in Figure 7. The results are thus not persuasive enough. Although some fault feature frequencies can be recognized, there are still many noise and modulation components.

**Figure 8:**
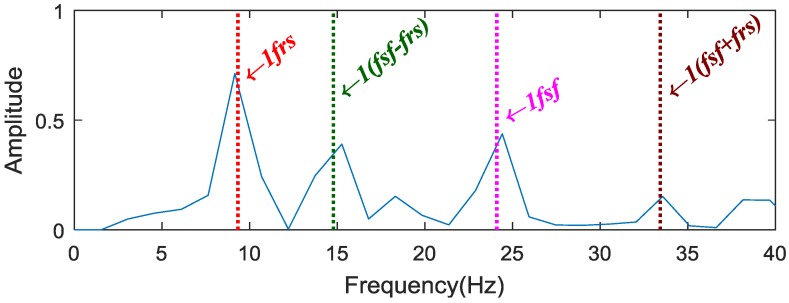
Results of the proposed method.

In order to solve the above problem, MSB is then applied to suppress the noise and decompose the modulated components. The normalized analysis results of vibration signal for the sun gear chipped based on the proposed method are shown in Figure 8: Some peaks correspond exactly to sun gear rotation frequency $f_{rs}$, sun gear characteristic frequency $f_{sf}$*,* and the combination $f_{sf}\pm f_{rs}$. The result demonstrates that the proposed method can get more effective and accurate fault features than the envelope analysis for sun gear fault diagnosis.

### 5.3. The Fault Diagnosis of the Planetary Gear Bearing

**Figure 9:**
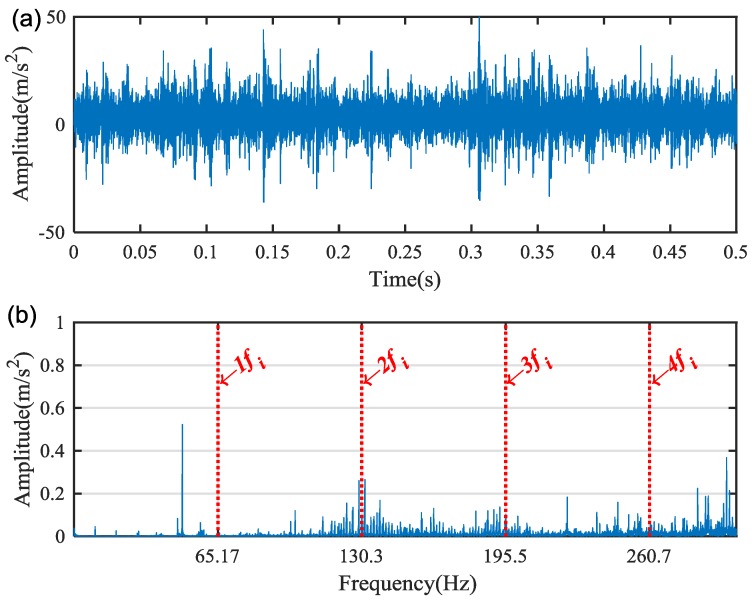
The raw vibration signal of the faulty bearing: (**a**) the waveform and (**b**) spectrum.

**Figure 10:**
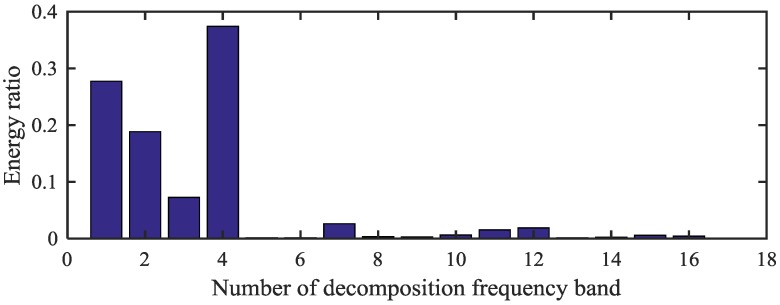
Energy distribution proportional histogram of the faulty bearing.

Figure 9a,b shows the waveform and spectrum of the measured vibration signal under the condition of planetary gear bearing with inner-race fault. It is difficult to identify the fault characteristic frequencies from the spectrum in Figure 9b. On the basis of the proposed method, the WPT is applied to decompose the vibration signal into a group of WP nodes with a complete binary tree form. Then the WPE matrix is calculated. The energy distribution proportional histogram of the vibration signal of the bearing inner-race fault is shown in Figure 10.

**Figure 11:**
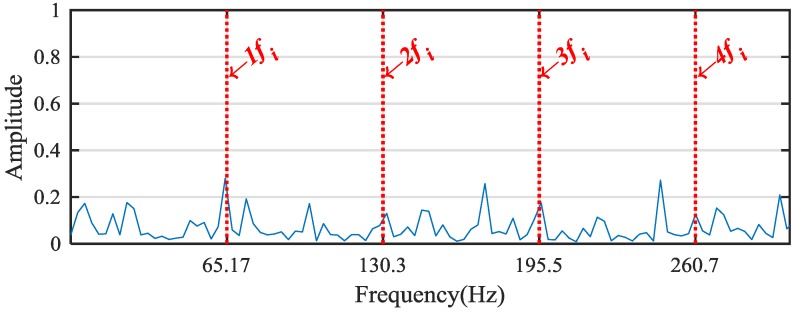
The envelope spectrum of the reconstructed signal.

**Figure 12:**
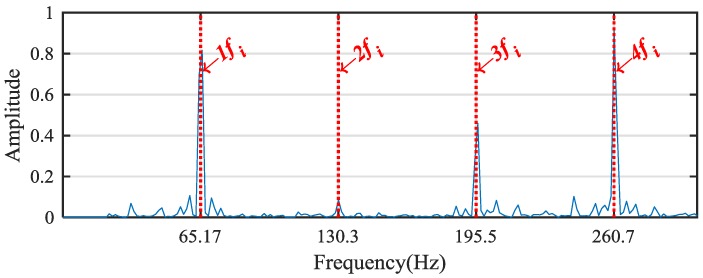
Results of the proposed method.

According to the WPE analysis results in Figure 10, ratios of the energy distribution at the node [4,0], [4,1], [4,2] and [4,3] are selected as the eigenvalues for the bearing inner-race fault signal. Then, the above four corresponding frequency bands are used to generate the reconstructed signal. Envelope analysis is then applied to analyze the fault signal; the corresponding normalized envelope spectrum is shown in Figure 11. It is obvious that the envelope analysis result does not present the characteristic frequencies of the bearing inner-race fault effectively owing to the strong noise interference. To deal with the problem, we took advantage of MSB to suppress noise and decompose modulations. The normalized results of the propose method is shown in Figure 12. It can be seen from the analysis results that the inner-race fault characteristic frequency $f_{i}$ and its harmonics can be clearly identified. In conclusion, the proposed method obtains more accurate results than the envelope analysis for the planetary gear bearing fault diagnosis.

## 6. Conclusions

This paper proposes a new analysis method based on WPE and MSB for planetary gearbox fault diagnosis. WPE reveals the energy distribution of the vibration signal in different time-frequency subspaces for the reconstructed signal obtained. MSB is used to enhance the reconstructed signal with relatively high energy vectors and highlight the fault features due to the effectiveness of MSB in demodulating modulation components and removing noise. Its efficiency has been evaluated on the experimental signals measured from planetary gearbox with sun gear chipped and planetary gear bearing with inner race fault. The results show that the proposed method is an effective and reliable method for early planetary gearbox faults diagnosis. However, the layers selection for the WPT and the spectral smearing of MSB would affect the diagnosis results of the proposed method. Hence, effective methods need to be studied to deal with the above issues in future works.

## Figures and Tables

**Table 1:** Structural parameters and kinematical parameters of the planetary gearbox.

Gear	Number of Teeth	Characteristic Frequency (Hz)	
**Sun gear**	10	24.10	$f_{sf}$
**Planet gears(number)**	26(3)	9.77	$f_{pf}$
**Ring gear**	62	3.89	$f_{rf}$

**Table 2:** Specification and characteristic frequency of the planetary gear bearing.

**Bearing Designation**	**Ball Numbers** **d (mm)**	**Pitch Diameter** $D_{m}$ **(mm)**	**Ball Number** **z**	**Contact Angle** **β**
	12	54	7.9	$0^{{}^{\circ}}$
**6008**	$f_{o}$	$f_{i}$	$f_{b}$	$f_{c}$
	49.25	65.17	33.60	4.10

**Table 3:** The frequency range of 4-layer wavelet packet decomposition.

Serial Number	Node Situation	Frequency/Hz
1st	node [4,0]	0–6000 Hz
2nd	node [4,1]	6000–12,000 Hz
3rd	node [4,2]	12,000–18,000 Hz
4th	node [4,3]	18,000–24,000 Hz
5th	node [4,4]	24,000–30,000 Hz
6th	node [4,5]	30,000–36,000 Hz
7th	node [4,6]	36,000–42,000 Hz
8th	node [4,7]	42,000–48,000 Hz
9th	node 4,8]	48,000–54,000 Hz
10th	node [4,9]	54,000–60,000 Hz
11th	node [4,10]	60,000–66,000 Hz
12th	node [4,11]	66,000–72,000 Hz
13th	node [4,12]	72,000–78,000 Hz
14th	node [4,13]	78,000–84,000 Hz
15th	node [4,14]	84,000–90,000 Hz
16th	node [4,15]	90,000–96,000 Hz
